# Supplementary material for: Ultrasmall metal alloy nanozymes mimicking neutrophil enzymatic cascades for tumor catalytic therapy
Source: Nat Commun. 2024 Feb 22;15:1626. doi: 10.1038/s41467-024-45668-3 (PMC10884023; doi:10.1038/s41467-024-45668-3)
Supplement: Supplementary file 1 — Supplementary Information [file 41467_2024_45668_MOESM1_ESM.pdf]

Supplementary Information

Ultrasmall metal alloy nanozymes mimicking neutrophil enzymatic cascades for tumor catalytic therapy

Xiangqin Meng[1,8], Huizhen Fan[1,8], Lei Chen[1,2,8], Jiuyang He[3,8], Chaoyi Hong[1,4], Jiaying Xie[1,4], Yinyin Hou[1,4], Kaidi Wang[1,4], Xingfa Gao[5], Lizeng Gao[1,4,6], Xiyun Yan[1,4,6,7*] & Kelong Fan[1,4,6,7*]

[1]*CAS Engineering Laboratory for Nanozyme, Key Laboratory of Biomacromolecules (CAS), CAS Center for Excellence in Biomacromolecules, Institute of Biophysics, Chinese Academy of Sciences, Beijing 100101, China*

[2]*Institute of Translational Medicine, Medical College, Yangzhou University, Yangzhou 225001, P. R. China*

[3]*Experimental Center of Advanced Materials, School of Materials Science & Engineering, Beijing Institute of Technology, Beijing 100081, P. R. China*

[4]*University of Chinese Academy of Sciences, Beijing 101408, P. R. China*

[5]*National Center for Nanoscience and Technology, Beijing 100190, P. R. China*

[6]*Nanozyme Medical Center, School of Basic Medical Sciences, Zhengzhou University, Zhengzhou 450052, P. R. China*

[7]*Nanozyme Laboratory in Zhongyuan, Zhengzhou, Henan, 451163, China*

[8]These authors contributed equally: Xiangqin Meng, Huizhen Fan, Lei Chen, Jiuyang He.

These authors jointly supervised this work: Xiyun Yan (email: yanxy@ibp.ac.cn), Kelong Fan (email: fankelong@ibp.ac.cn).

Supplementary Figures 1 to 20.

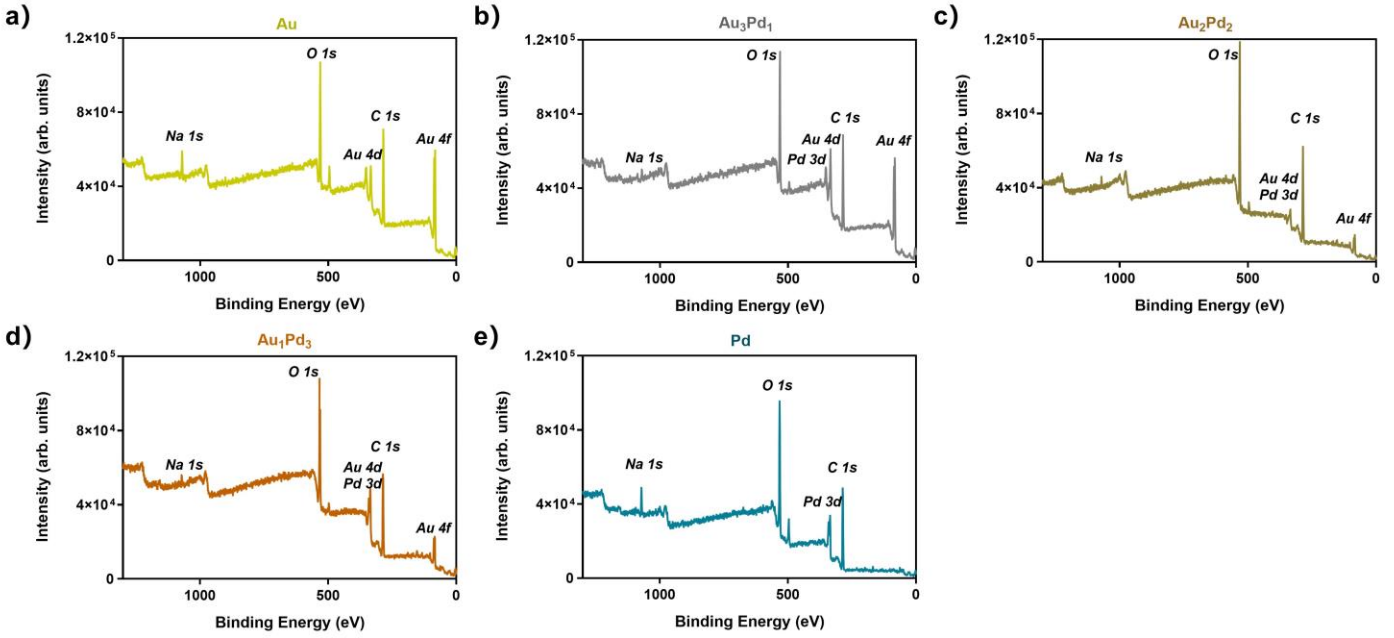

Supplementary Fig. 1 The XPS spectra of five AuPd alloy nanozymes. Source data are provided as a Source Data file.

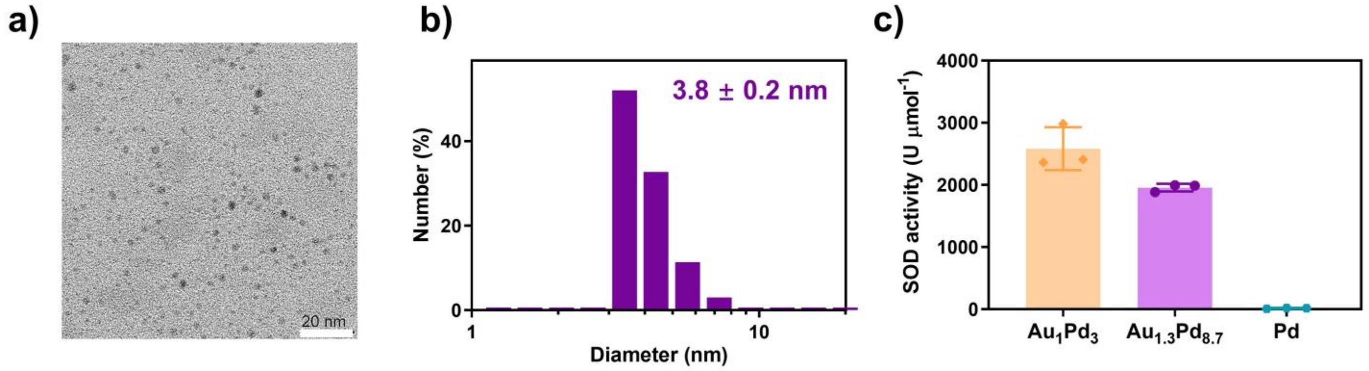

Supplementary Fig. 2 The (a) TEM image, (b) hydrodynamic diameter distribution and (c) SOD-like activity (n = 3 independent experiments) of the $Au_{1.3}Pd_{8.7}$ nanozymes. Scale bar = 20 nm. All data are presented as mean ± STD. Source data are provided as a Source Data file.

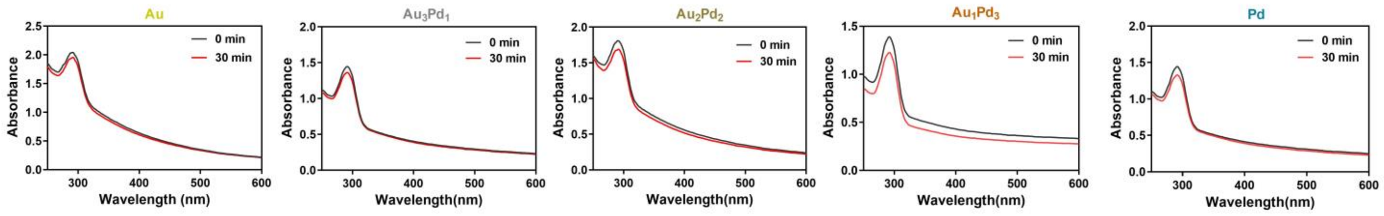

Supplementary Fig. 3 UV/Vis spectra of MCD after treated with five AuPd alloy nanozymes. Source data are provided as a Source Data file.

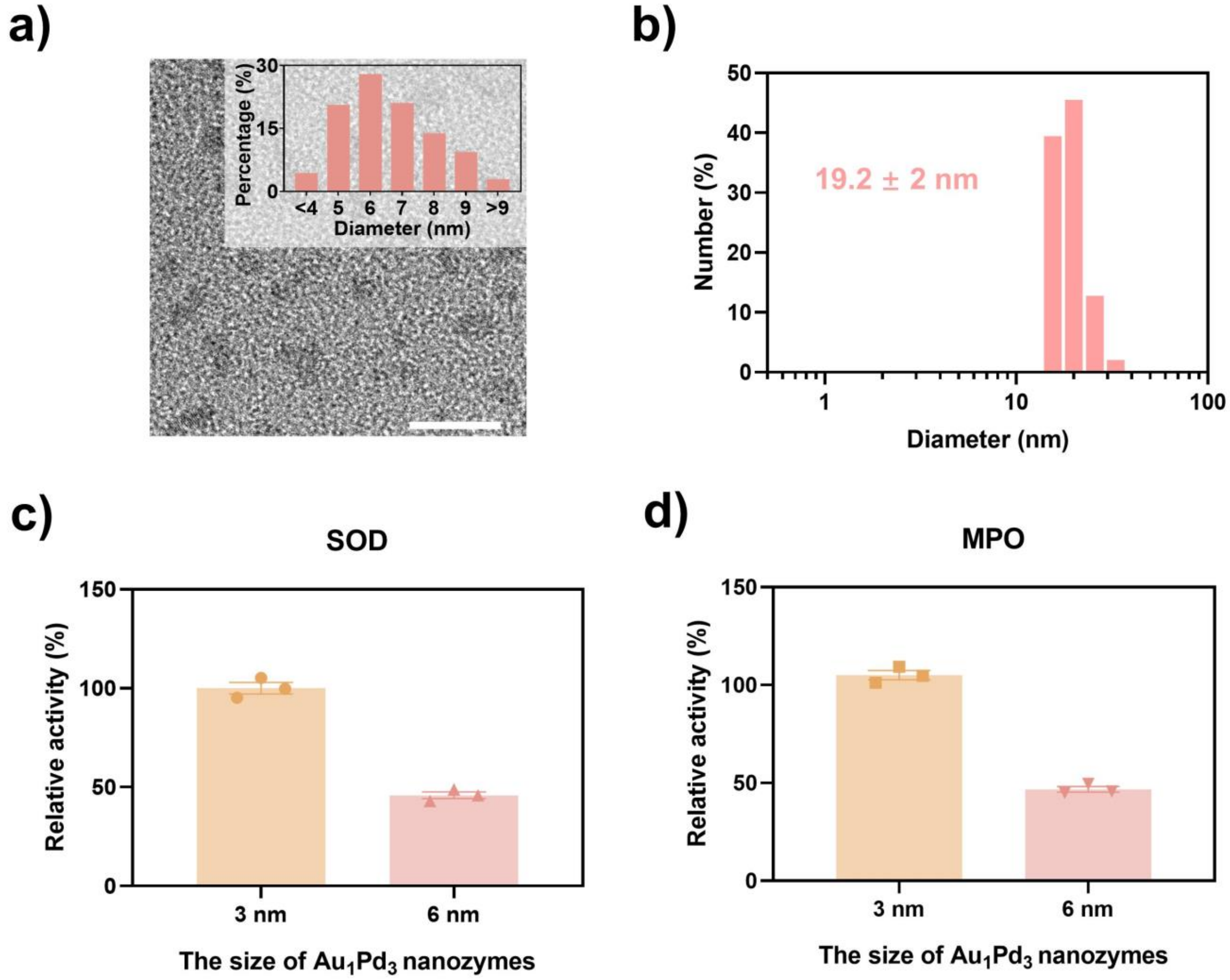

Supplementary Fig. 4 The (a) TEM image and size distribution, (b) hydrodynamic diameter distribution, (c) SOD-like activity (n = 3 independent experiments) and (d) MPO-like activity (n = 3 independent experiments) of large-sized Au_1Pd_3 alloy nanozymes. Scale bar = 10 nm. All data are presented as mean ± STD. Source data are provided as a Source Data file.

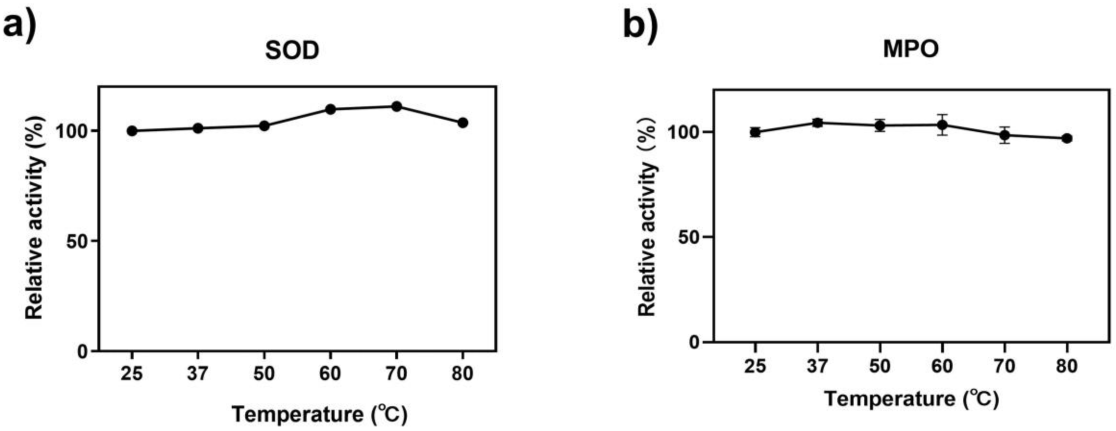

Supplementary Fig. 5 The (a) SOD-like activity (n = 3 independent experiments) and (b) MPO-like activity (n = 3 independent experiments) of Au_1Pd_3 alloy nanozymes after incubation at different temperatures for 2 hours. All data are presented as mean ± STD. Source data are provided as a Source Data file.

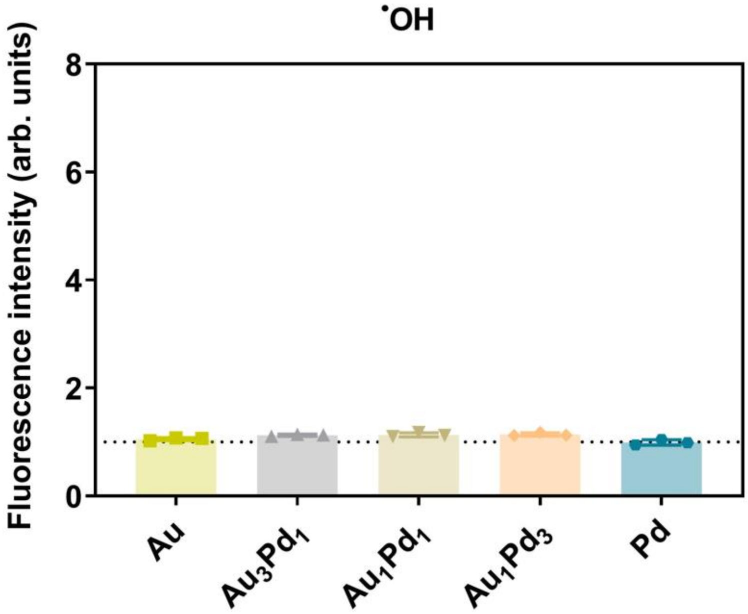

Supplementary Fig. 6 Production of $OH^{•}$ detected by HPF probe of the SOD-MPO-like cascade activity (n = 3 independent experiments). All data are presented as mean ± STD. Source data are provided as a Source Data file.

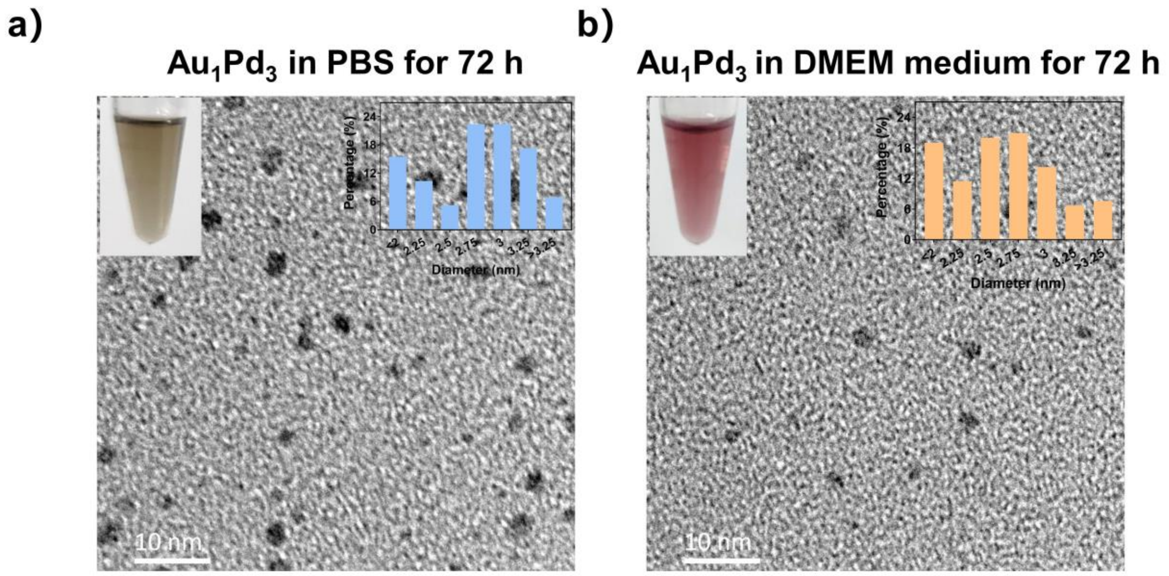

Supplementary Fig. 7 TEM images, pictures and size distribution and of Au_1Pd_3 nanozymes in (a) PBS and (b) DMEM medium for 72 h. Scale bar = 10 nm. Source data are provided as a Source Data file.

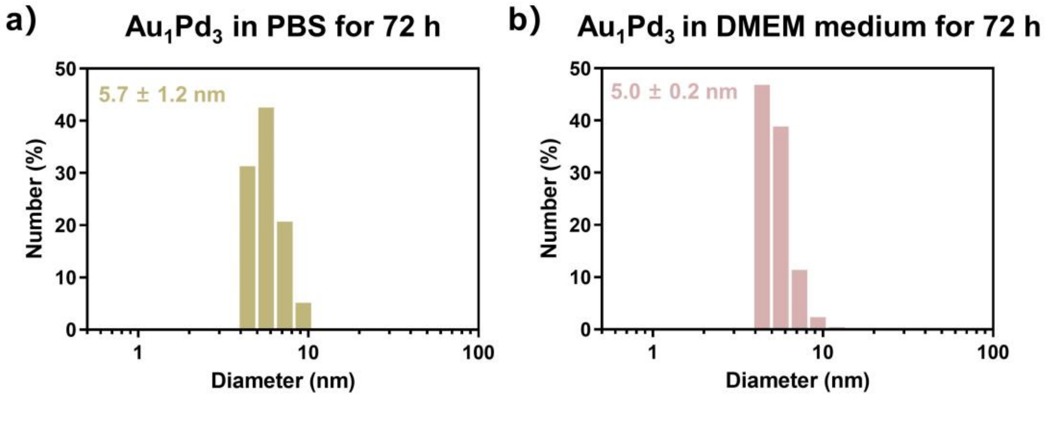

Supplementary Fig. 8 Hydrodynamic diameter distribution of Au_1Pd_3 nanozymes in (a) PBS and (b) DMEM medium for 72 h. Source data are provided as a Source Data file.

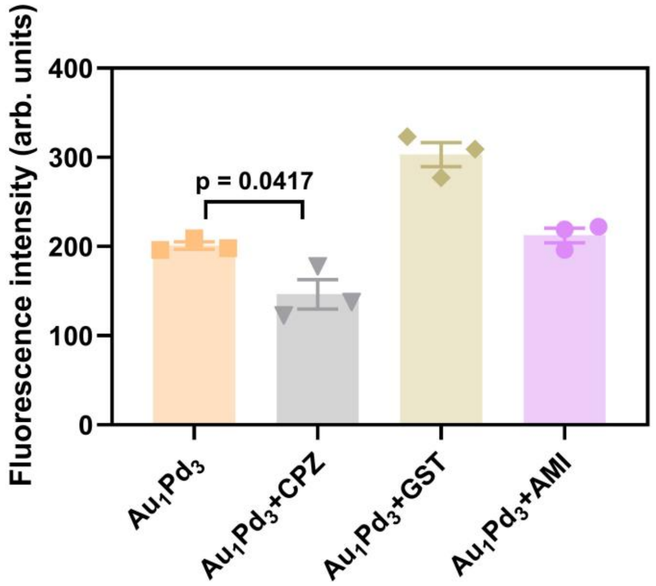

Supplementary Fig. 9 Cellular uptake of Au_1Pd_3 nanozymes in CT26 cells quantified by flow cytometry in the presence of different endocytosis inhibitors (n = 3 independent experiments). *P* values are determined with one-way ANOVA Tukey's multiple comparisons test. All data are presented as mean ± STD. Source data are provided as a Source Data file.

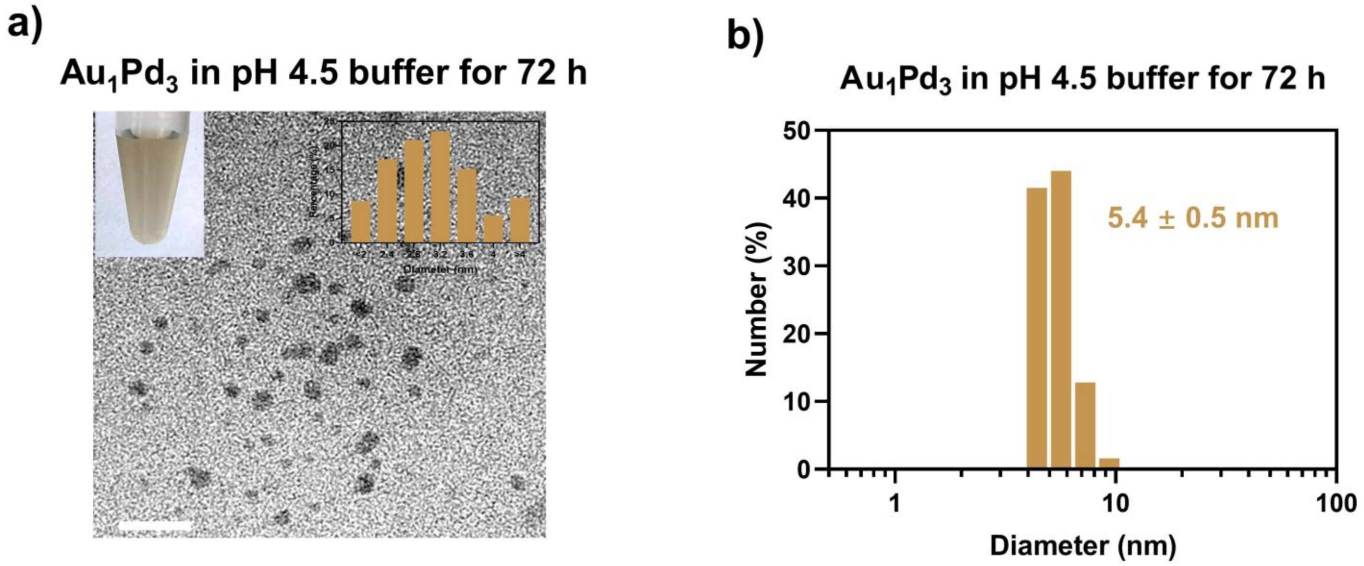

Supplementary Fig. 10 The (a) TEM image, picture and size distribution and (b) hydrodynamic diameter distribution of Au_1Pd_3 nanozymes in a pH 4.5 lysosome-mimicking environment for 72 h. Scale bar = 10 nm. Source data are provided as a Source Data file.

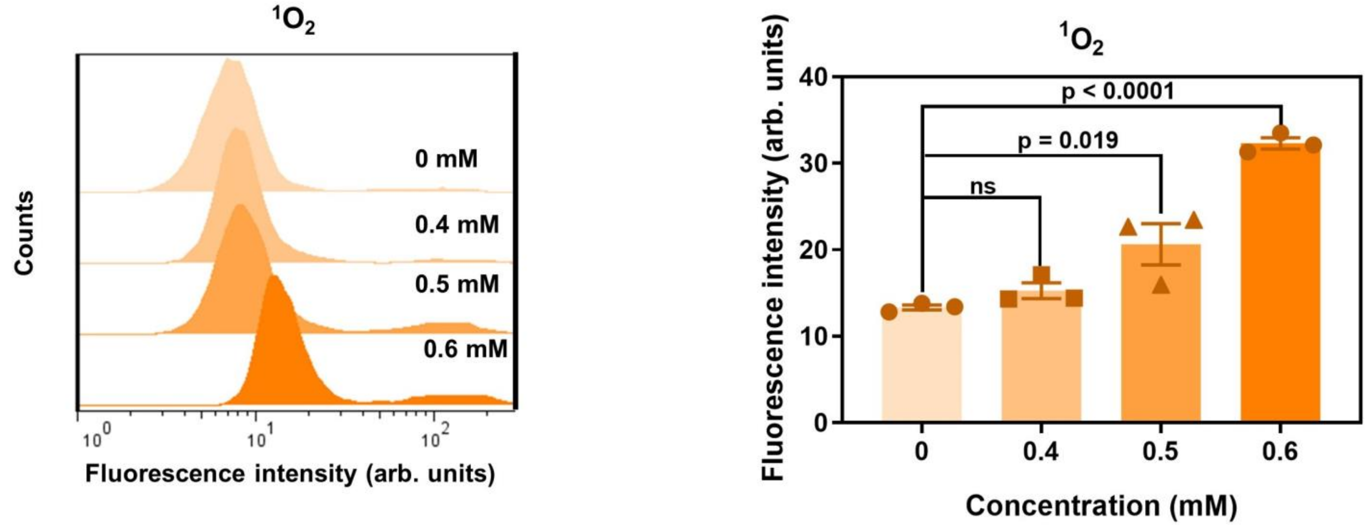

Supplementary Fig. 11 Flow cytometry analyses (left) and the corresponding data analyses (right) of CT26 cells treated with Au_1Pd_3 nanozymes using SOSG as detector for $^1O_2$ (n = 3 independent experiments). *P* values are determined with one-way ANOVA Tukey's multiple comparisons test. All data are presented as mean ± STD. Source data are provided as a Source Data file.

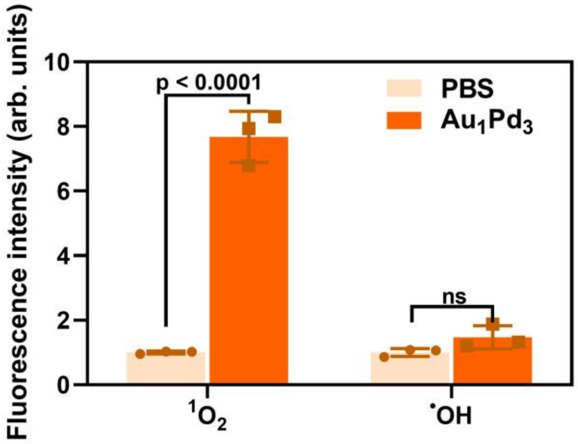

Supplementary Fig. 12 Flow cytometry data analyses of CT26 cells treated with Au_1Pd_3 nanozymes using SOSG and HPF as detector for $^1O_2$ and $OH^•$, respectively (n = 3 independent experiments). *P* values are determined with two-way ANOVA Sidak's multiple comparisons test. All data are presented as mean ± STD. Source data are provided as a Source Data file.

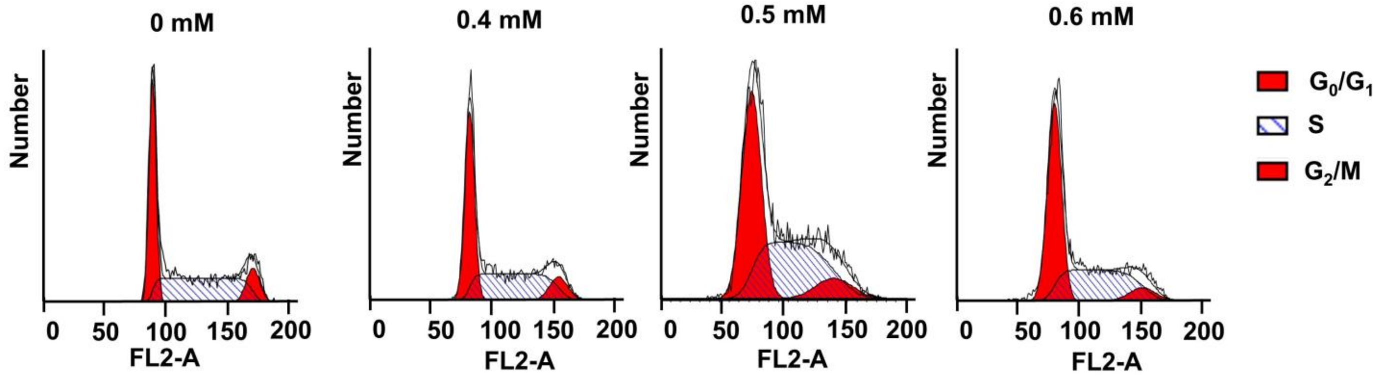

Supplementary Fig. 13 Effect of Au_1Pd_3 nanozymes on the cell cycles of CT26 cells.

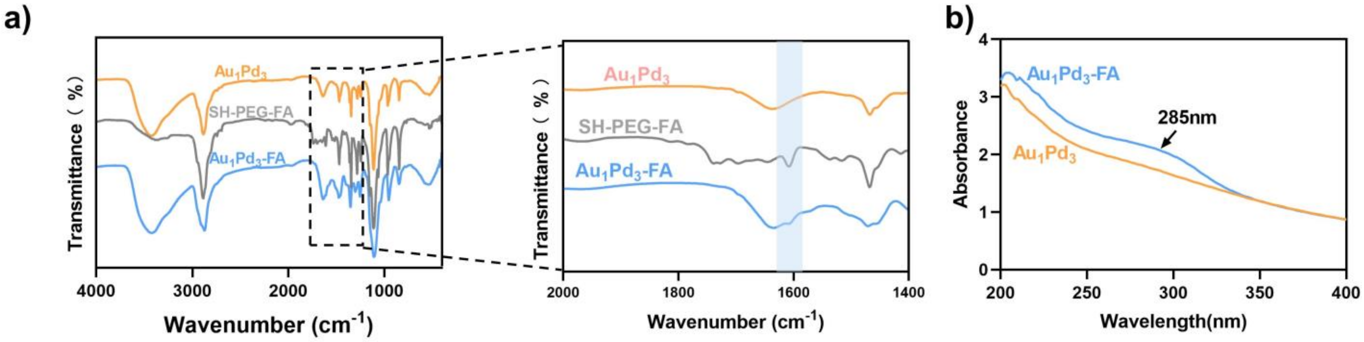

Supplementary Fig. 14 (a) FTIR spectra of Au_1Pd_3 nanozymes, Au_1Pd_3-FA nanozymes and SF-PEG-FA. (b) UV/Vis spectra of Au_1Pd_3 and Au_1Pd_3-FA nanozymes. Source data are provided as a Source Data file.

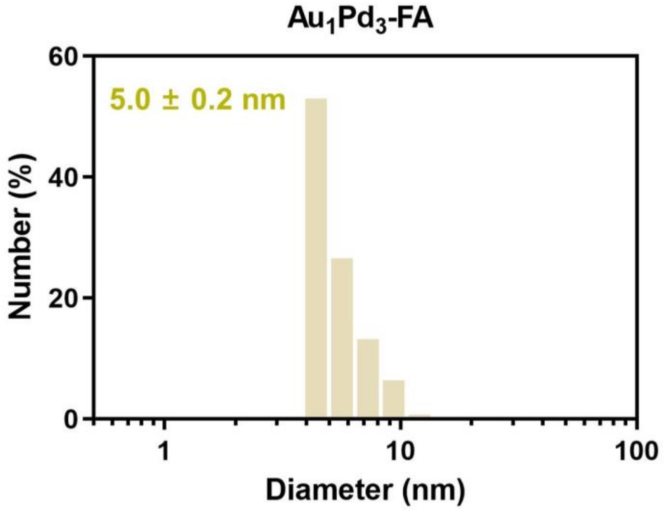

Supplementary Fig. 15 Hydrodynamic diameter distribution of Au_1Pd_3-FA nanozymes. Source data are provided as a Source Data file.

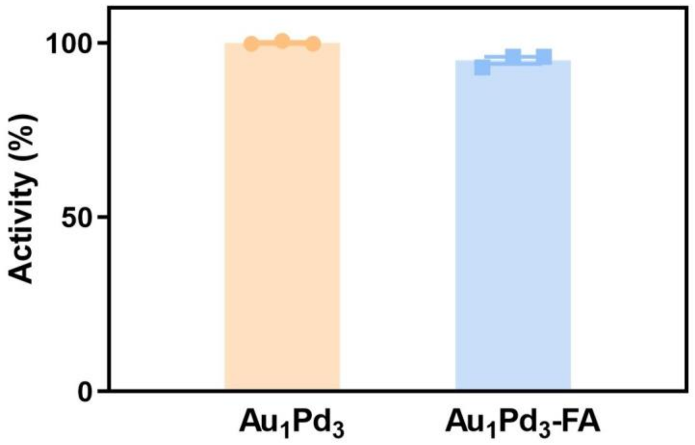

Supplementary Fig. 16 The SOD-MPO-like cascade activities of Au_1Pd_3 and Au_1Pd_3-FA nanozymes (n = 3 independent experiments). All data are presented as mean ± STD. Source data are provided as a Source Data file.

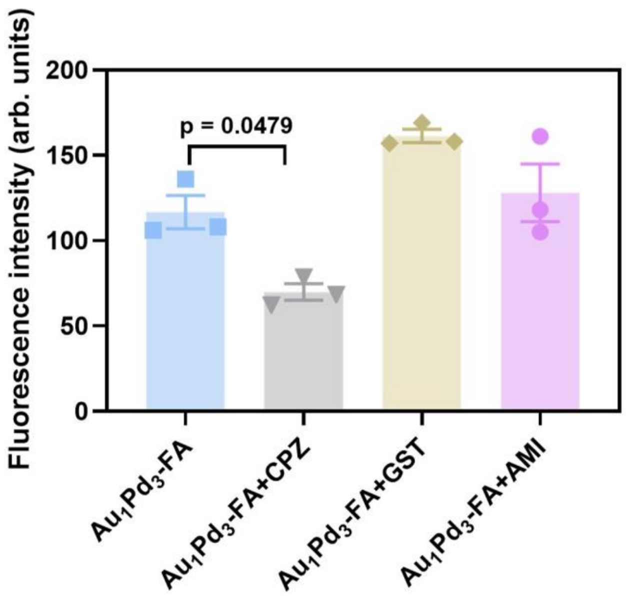

Supplementary Fig. 17 Cellular uptake of Au_1Pd_3 nanozymes in CT26 cells quantified by flow cytometry in the presence of different endocytosis inhibitors (n = 3 independent experiments). *P* values are determined with one-way ANOVA Tukey's multiple comparisons test. All data are presented as mean ± STD. Source data are provided as a Source Data file.

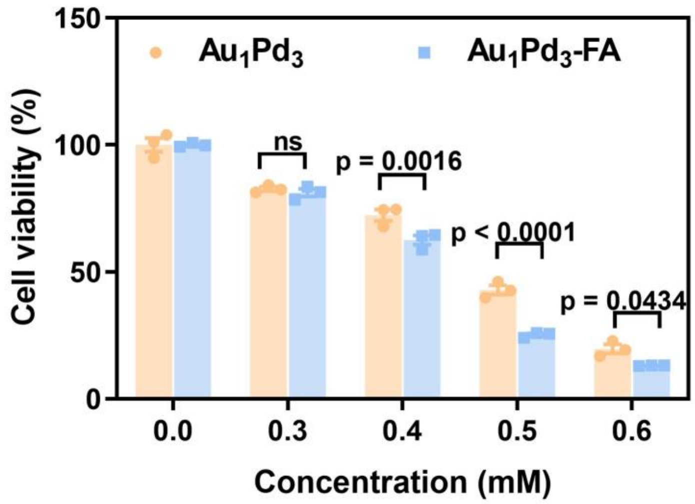

Supplementary Fig. 18 Effect of Au_1Pd_3 and Au_1Pd_3-FA nanozymes on CT26 cell viability (n = 3 independent experiments). *P* values are determined with two-way ANOVA Sidak's multiple comparisons test. All data are presented as mean ± STD. Source data are provided as a Source Data file.

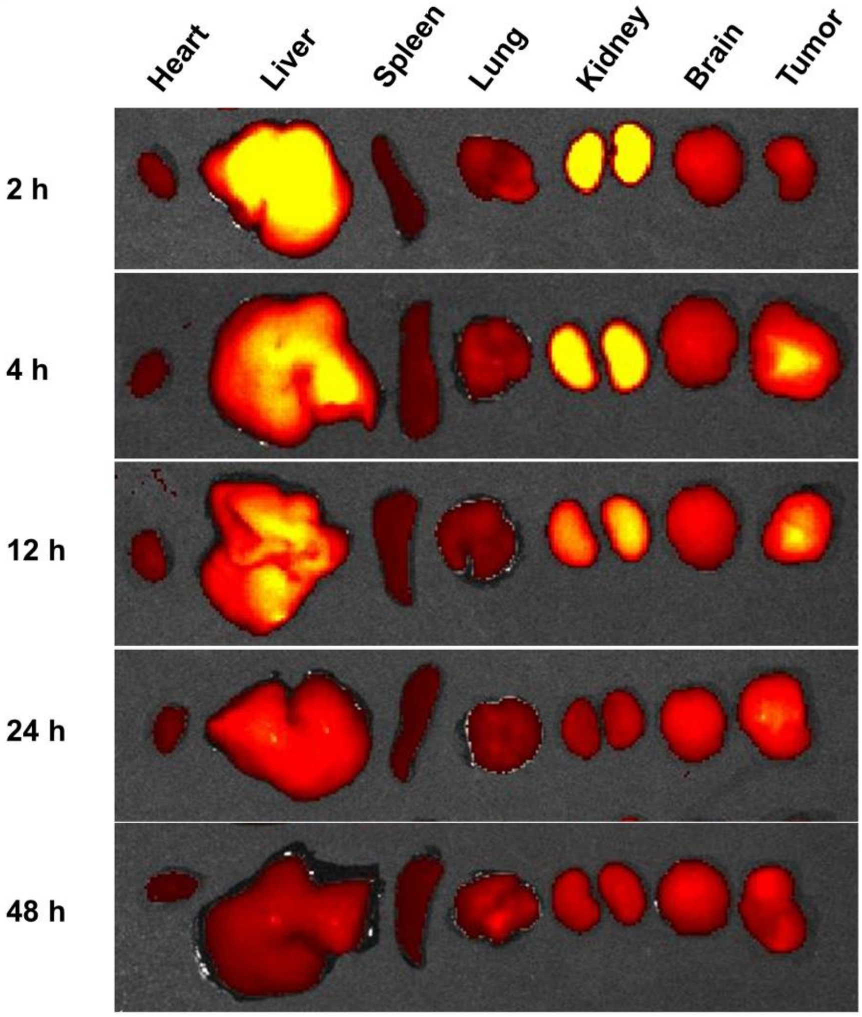

Supplementary Fig. 19 The representative images of the biodistribution of Au_1Pd_3-FA-Cy5.5 nanozymes in main organs and tumors at different time points (n = 3 mice per group).

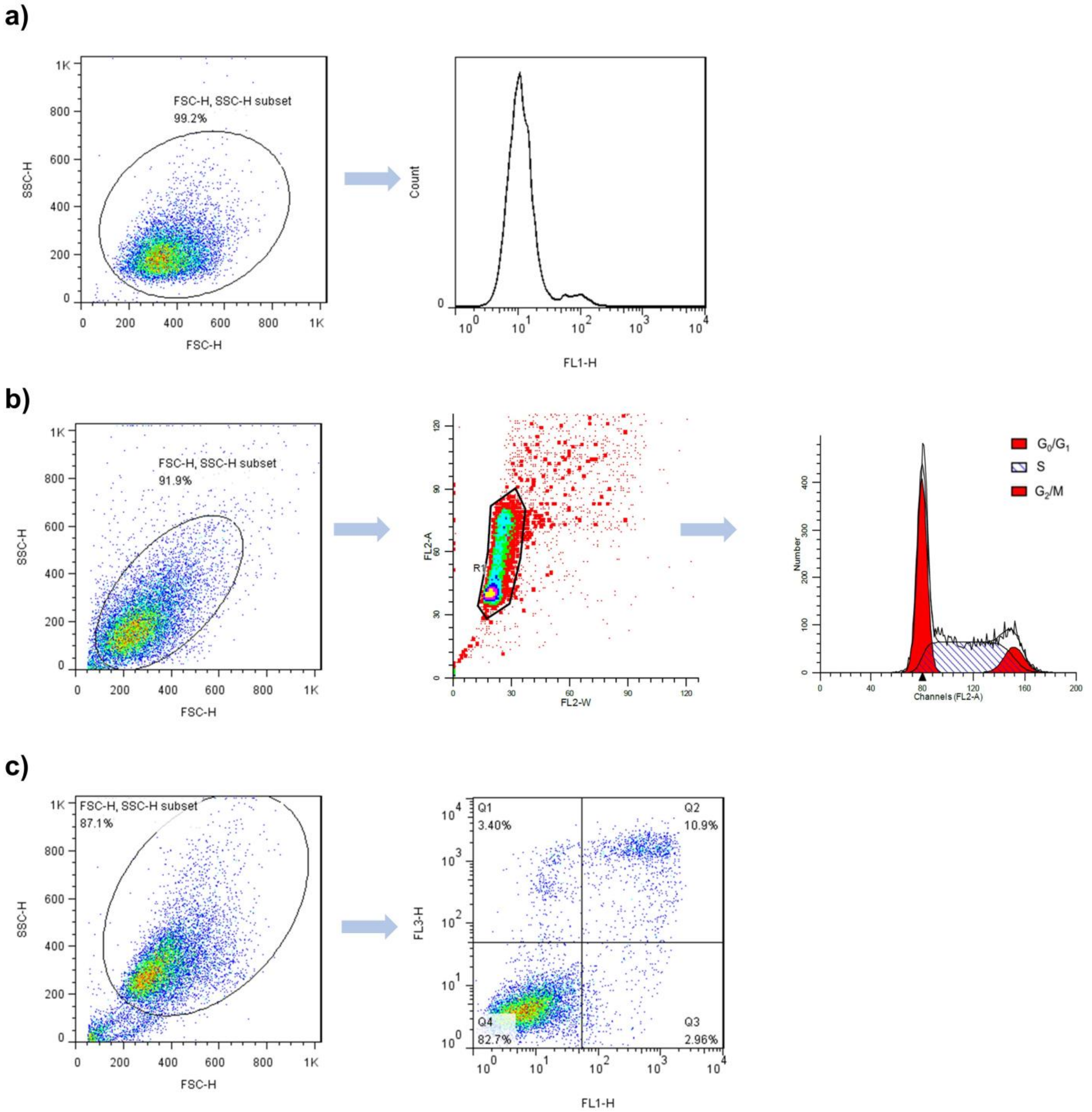

Supplementary Fig. 20 (a) Flow cytometry gating strategies for experiments in Figs. 2g, 2i and Supplementary Figs. 6-8, 13. (b) Flow cytometry gating strategies for experiments in Fig. 5c and Supplementary Fig. 9. (c) Flow cytometry gating strategies for experiments in Figs. 5d-e.

Supplementary Tables

Supplementary Table 1 The adsorption energy of $HO_2^{\bullet}$ free radicals on (111) facets of Au, Pd and their alloys, the red value is the adsorption energy in the lowest energetic conformation.

	top	bridge	fcc	hcp
Au (111)	- 0.46	- 0.47	- 0.42	- 0.42
Au_3Pd_1 (111)	- 0.96		- 0.76	- 0.66
Au_2Pd_2 (111)	- 1.15	- 1.00	- 1.00	
Au_1Pd_3 (111)	- 1.61	- 1.59	- 1.49	- 1.53
Pd (111)	- 1.30	- 1.22	- 1.17	- 1.18

Supplementary Methods

First-principles calculations

First principle density functional theory (DFT) calculations had been done using Vienna ab initio Simulation Package (VASP.6.1.0)[1-3]. The electron-ion interactions were described by using projector augmented wave (PAW) method[4]. The generalized gradient approximation (GGA) was used with the exchange-correlation functional of Perdew-Burke−Ernzerhof (PBE)[5]. All geometry optimizations and energy calculations were performed in a plane-wave basis set up to an energy cutoff of 400 eV with a first-order Methfessel−Paxton[6] smearing of 0.2 eV. We employed a four-layered slab in (111) direction with a (3×3) unit cell in the lateral direction to model the Au (111) and Pd (111) surface. To separate the slab from its periodic images to avoid spurious interaction, a vacuum height of 15 Å along the vertical direction was selected. The calculations were performed using the (2 × 2 × 1) Monkhorst-Pack mesh kpoints[7] for (2 × 2) unit cell. Upon geometry optimization, the top two layers of Au (111) and Pd (111) were fully relaxed and the bottom layers were kept fixed. In tailored $Au_{4-x}Pd_x$ bulk alloys, the contents of Pd were 25%, 50% and 75%, respectively. Consequently, the bulk alloys of Au_3Pd_1, Au_2Pd_2, and Au_1Pd_3 were obtained. And Au_3Pd_1 (111), Au_2Pd_2 (111) and Au_1Pd_3 (111) surfaces were also cleaved in the similar method of Au (111) surface with (4×4) unit cells. Conjugated-gradient algorithm was used to optimize the structures. In all calculations, the convergence criterion of electronic structures was set to 10^{-6} eV, and the atomic positions were allowed to relax until the forces were less than 0.02 eV $Å^{-1}$.

The d-band center for the (111) facets of Au, Pd and their alloys can be determined using the following formula:

$$\varepsilon_d = \frac{\int_{-\infty}^{\infty} n_d(\varepsilon)\varepsilon d\varepsilon}{\int_{-\infty}^{\infty} n_d(\varepsilon) d\varepsilon}$$

where Ɛ is the d-band energy and n(Ɛ) is the d-band density. In this article, the d-band center of (111) facets is

the average of surface Au and Pd atoms.

The adsorption energies were calculated in the following expressions:

$E_{ads} = E_{slab+mol} - (E_{slab} + E_{mol})$

where $E_{slab+mol}$ represents the total energies of the chosen surface with adsorbate on it, and the E_{slab} and E_{mol} denote the bare chosen surface and the adsorbate, respectively. The “climbing images” nudged elastic band (CI-NEB) algorithm[8] was employed to search for transition states (TSs). For the search of TSs, the same force threshold as the geometrical optimization was used. Stretching frequencies were analyzed to characterize the transition states, for which only one imaginary frequency was found for each of them[9]. To investigate the dissociative adsorption and rearrangement of $HO_2^\bullet$ radicals on metal surfaces, spin polarized PBE functionals were used for the calculations, because $HO_2^\bullet$ radical has a doublet ground state. For pure metal surfaces, only spin unpolarized functional was used.

To evaluate the thermal stability of two $HO_2^\bullet$ radical on Au_1Pd_3 (111) and Pd (111) surfaces at 300 K, the AIMD simulations were carried out using a canonical ensemble with a Nosé−Hoover heat bath scheme[10]. The simulation time was 20 ps.

Supplementary References

1. Kresse, G. & Joubert, D. From ultrasoft pseudopotentials to the projector augmented-wave method. *Physical Review B* **59**, 1758-1775 (1999).
2. Kresse, G. & Furthmuller, J. Efficiency of ab-initio total energy calculations for metals and semiconductors using a plane-wave basis set. *Computational Materials Science* **6**, 15-50 (1996).
3. Kresse, G. & Furthmuller, J. Efficient iterative schemes for ab initio total-energy calculations using a plane-wave basis set. *Physical Review B* **54**, 11169-11186 (1996).
4. Blochl, P. E. Projector augmented-wave method. *Physical Review B* **50**, 17953-17979 (1994).
5. Perdew, J. P., Burke, K. & Ernzerhof, M. Generalized gradient approximation made simple. *Physical Review Letters* **77**, 3865-3868 (1996).
6. Methfessel, M. & Paxton, A. T. High-precision sampling for brillouin-zone integration in metals. *Physical Review B* **40**, 3616-3621 (1989).
7. Monkhorst, H. J. & Pack, J. D. Special points for brillouin-zone integrations. *Physical Review B* **13**, 5188-5192 (1976).
8. Henkelman, G., Uberuaga, B. P. & Jonsson, H. A climbing image nudged elastic band method for finding saddle points and minimum energy paths. *Journal of Chemical Physics* **113**, 9901-9904 (2000).
9. Sheppard, D., Terrell, R. & Henkelman, G. Optimization methods for finding minimum energy paths. *Journal of Chemical Physics* **128**, 2008).
10. Nose, S. A unified formulation of the constant temperature molecular-dynamics methods. *Journal of Chemical Physics* **81**, 511-519 (1984).
