# Supplementary material for: Ultrasmall metal alloy nanozymes mimicking neutrophil enzymatic cascades for tumor catalytic therapy
Source: Nat Commun. 2024 Feb 22;15:1626. doi: 10.1038/s41467-024-45668-3 (PMC10884023; doi:10.1038/s41467-024-45668-3)
Supplement: Supplementary file 2 — Reporting Summary [file 41467_2024_45668_MOESM2_ESM.pdf]

nature portfolio

Corresponding author(s): Kelong Fan

Last updated by author(s): Jan 30, 2024

Reporting Summary

Nature Portfolio wishes to improve the reproducibility of the work that we publish. This form provides structure for consistency and transparency in reporting. For further information on Nature Portfolio policies, see our Editorial Policies and the Editorial Policy Checklist.

Statistics

For all statistical analyses, confirm that the following items are present in the figure legend, table legend, main text, or Methods section.

n/a	Confirmed	
☐	☒	The exact sample size (*n*) for each experimental group/condition, given as a discrete number and unit of measurement
☐	☒	A statement on whether measurements were taken from distinct samples or whether the same sample was measured repeatedly
☐	☒	The statistical test(s) used AND whether they are one- or two-sided<br>*Only common tests should be described solely by name; describe more complex techniques in the Methods section.*
☒	☐	A description of all covariates tested
☒	☐	A description of any assumptions or corrections, such as tests of normality and adjustment for multiple comparisons
☐	☒	A full description of the statistical parameters including central tendency (e.g. means) or other basic estimates (e.g. regression coefficient) AND variation (e.g. standard deviation) or associated estimates of uncertainty (e.g. confidence intervals)
☐	☒	For null hypothesis testing, the test statistic (e.g. *F*, *t*, *r*) with confidence intervals, effect sizes, degrees of freedom and *P* value noted<br>*Give P values as exact values whenever suitable.*
☒	☐	For Bayesian analysis, information on the choice of priors and Markov chain Monte Carlo settings
☒	☐	For hierarchical and complex designs, identification of the appropriate level for tests and full reporting of outcomes
☒	☐	Estimates of effect sizes (e.g. Cohen's *d*, Pearson's *r*), indicating how they were calculated

Our web collection on statistics for biologists contains articles on many of the points above.

Software and code

Policy information about availability of computer code

Data collection	HRTEM images were obtained using a FEI Tecnai G2 F30 transmission electron microscope. The FTIR spectra were collected using a Nicolet™ iS™ 10 FTIR spectrometer. The Raman spectra were obtained using a Renishaw inVia Qontor instrument with 532 nm excitation. ICP-OES measurements were collected using an Agilent ICP-OES 730 instrument. ICP-MS measurements were collected using an Agilent ICP-MS 7800 instrument. XRD patterns were obtained using a Bruker D8 Advance X-ray powder diffractometer with Cu Kα radiation. XPS experiments were performed using a Thermo escalab 250Xi spectrometer. DLS experiments were carried out using a Wyatt DynaPro NanoStar 271-DPN dynamic light scattering equipment. TEM images were obtained with a FEI Tecnai Spiritelectron microscope operated at 120 kV. Flow cytometry data was collected using the FACS CaliburTM instrument from Becton Dickinson (USA). Confocal images were obtained using a ZIESS-LSM700 (Germany). Fluorescence imaging was carried out using an in vivo imaging instrument (IVIS Lumina 3, PE, USA).
Data analysis	The particle sizes in HRTEM images were calculated using Image Pro Plus software. The flow cytometry results were analyzed using FlowJo 7.6 software. The confocal images were analyzed by ZEN 2012 software. The in vivo fluorescence images were analyzed through the IVIS Living Image 3.0 software (PerkinElmer, USA). General statistical data were analyzed by Graphpad prism 8.

For manuscripts utilizing custom algorithms or software that are central to the research but not yet described in published literature, software must be made available to editors and reviewers. We strongly encourage code deposition in a community repository (e.g. GitHub). See the Nature Portfolio guidelines for submitting code & software for further information.

Data

Policy information about availability of data

All manuscripts must include a data availability statement. This statement should provide the following information, where applicable:

- Accession codes, unique identifiers, or web links for publicly available datasets
- A description of any restrictions on data availability
- For clinical datasets or third party data, please ensure that the statement adheres to our policy

Data supporting the findings of this work are available with the paper and its Supplementary information files. Source data are provided with this paper and the raw data are available upon request to the corresponding authors.

Research involving human participants, their data, or biological material

Policy information about studies with human participants or human data. See also policy information about sex, gender (identity/presentation), and sexual orientation and race, ethnicity and racism.

Reporting on sex and gender	N/A
Reporting on race, ethnicity, or other socially relevant groupings	N/A
Population characteristics	N/A
Recruitment	N/A
Ethics oversight	N/A

Note that full information on the approval of the study protocol must also be provided in the manuscript.

Field-specific reporting

Please select the one below that is the best fit for your research. If you are not sure, read the appropriate sections before making your selection.

[x] Life sciences [ ] Behavioural & social sciences [ ] Ecological, evolutionary & environmental sciences

For a reference copy of the document with all sections, see nature.com/documents/nr-reporting-summary-flat.pdf

Life sciences study design

All studies must disclose on these points even when the disclosure is negative.

Sample size	Sample size choice in the manuscript was consistent with previous studies (ref. He, X., Zhang, L., Queme, L. et al, 2018. http://doi.org/10.1038/nm.4438; Li, F., Sun, H., Ren, J. et al, 2022.http://doi.org/10.1038/s41467-022-35022-w). Exact sample size for each experiment is shown in the figure legend.
Data exclusions	No data was excluded from the analysis.
Replication	Replicates of at least 3 were used for all experiments. We confirmed that the attempts at replication were successful. Each figure contains detailed experimental replicates in the figure.
Randomization	All samples/organisms were randomly allocated into experimental groups.
Blinding	Investigators were not blinded for nanomaterial synthesis. For all other experiments, investigators were blinded to group allocation during data collection and analysis.

Reporting for specific materials, systems and methods

We require information from authors about some types of materials, experimental systems and methods used in many studies. Here, indicate whether each material, system or method listed is relevant to your study. If you are not sure if a list item applies to your research, read the appropriate section before selecting a response.

Materials & experimental systems

n/a	Involved in the study	
☐	☒	Antibodies
☐	☒	Eukaryotic cell lines
☒	☐	Palaeontology and archaeology
☐	☒	Animals and other organisms
☒	☐	Clinical data
☒	☐	Dual use research of concern
☒	☐	Plants

Methods

n/a	Involved in the study	
☒	☐	ChIP-seq
☐	☒	Flow cytometry
☒	☐	MRI-based neuroimaging

Antibodies

Antibodies used	Anti-γ H2AX-antibody was used as an ingredient in commercial DNA Damage Assay Kit by γ H2AX Immunofluorescence (C2035 S, Beyotime Biotechnology, Shanghai, China).
Validation	https://www.beyotime.com/product/C2035S.htm

Eukaryotic cell lines

Policy information about cell lines and Sex and Gender in Research

Cell line source(s)	CT26 cell line and 4T1 cell lines were purchased from ATCC. AML12 cell line was purchased from Hunan Fenghui Biotechnology Co., Ltd (China).
Authentication	A short tandem repeat DNA profiling method was used to authenticate the cell lines and the results were compared with reference database.
Mycoplasma contamination	The cell lines were tested as mycoplasma negative by a standard PCR protocols.
Commonly misidentified lines (See ICLAC register)	No commonly misidentified cell lines were used in this study.

Animals and other research organisms

Policy information about studies involving animals; ARRIVE guidelines recommended for reporting animal research, and Sex and Gender in Research

Laboratory animals	Balb/c mice, female, 6-8 weeks. All mice were group-housed 5 mice per cage in temperature (22-26℃) and humidity (40%-70%) house rooms on a 12h light, 12h dark cycle.
Wild animals	The study did not involve wild animals.
Reporting on sex	Theoretically, the findings are applicable to both females and males. Only female mice were used for the in vivo experiments in this study.
Field-collected samples	Field-collected samples were not involved in this study.
Ethics oversight	The animal studies were conducted in accordance with the approved protocol of the Institutional Animal Care and Use Committee (IACUC) of the Institute of Biophysics, Chinese Academy of Sciences (Project number: SYXK2023168). The IACUC permits a maximum tumor size of 15 mm in diameter, in our work no mice exceeded this criterion. According to the IACUC guidelines, mice that experience weight loss exceeding 20% or display symptoms such as hunched posture, impaired locomotion or respiratory distress should be promptly euthanized using CO2 gas. Otherwise, the mice were euthanized at the conclusion of the experiment.

Note that full information on the approval of the study protocol must also be provided in the manuscript.

Flow Cytometry

Plots

Confirm that:

☒ The axis labels state the marker and fluorochrome used (e.g. CD4-FITC).

☒ The axis scales are clearly visible. Include numbers along axes only for bottom left plot of group (a 'group' is an analysis of identical markers).

☒ All plots are contour plots with outliers or pseudocolor plots.

☒ A numerical value for number of cells or percentage (with statistics) is provided.

Methodology

Sample preparation	Initially, CT26 cells were plated in six-well plates at a density of 150000 cells/well and allowed to adhere overnight. Various concentrations of Au1Pd3 nanozymes were then added to the wells for 24 h of incubation. Afterward, the medium was removed, and the cells were detached and washed with a PBS buffer. Next, the cells were treated with APF probe, HPF probe, PI and RNase solution or Annexin V-FITC and PI solution at 37°C for 30 min, followed by another PBS buffer rinse.
Instrument	FACS CaliburTM, Becton Dickinson, Franklin Lakes, NJ, USA
Software	FlowJo 7.6 software
Cell population abundance	10^8-10^9 cells/mL analyzed by cellometer auto t4
Gating strategy	Cells were gated based on size and granularity of forward and side scatter (SSC and FSC) and the cell gate is analyzed for specific fluorescence.

[x] Tick this box to confirm that a figure exemplifying the gating strategy is provided in the Supplementary Information.
